# Neutrophilic dermatosis of the dorsal hand with sporotrichoid spread

**DOI:** 10.1016/j.jdcr.2025.07.001

**Published:** 2025-07-15

**Authors:** Ella Engels, Jessica Forman, Christopher T. Richardson

**Affiliations:** a University of Rochester School of Medicine and Dentistry, Rochester, New York; b Department of Dermatology, University of Rochester Medical Center, Rochester, New York

**Keywords:** acute febrile neutrophilic dermatosis, neutrophilic dermatosis, sporotrichoid, Sweet's syndrome

## Introduction

Acute febrile neutrophilic dermatosis (Sweet's syndrome) is a serious, noninfectious dermatologic condition that presents with painful, erythematous skin lesions often accompanied by systemic symptoms, including fever, joint pain, and headache. Histopathology characteristically shows a neutrophilic infiltrate in the upper dermis.^1^^,^^2^ Neutrophilic dermatosis of the dorsal hands (NDDH) is a localized variant of Sweet's syndrome and commonly occurs in patients with underlying hematologic malignancy.^3^, ^4^, ^5^ Here, we discuss a patient with myelodysplastic syndrome (MDS) that developed NDDH with lesions progressing in a sporotrichoid pattern, a distribution rarely reported in Sweet's syndrome and more typically concerning for an infectious etiology.^1^

## Case report

**Fig 1:**
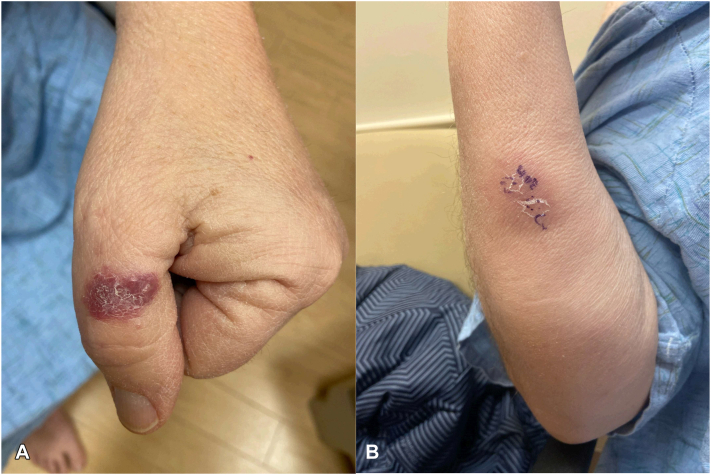
Rash at initial presentation. Pink indurated papulonodules involving the **(A)** left thumb and **(B)** right forearm at initial dermatologic evaluation. Biopsy sites noted.

A 66-year-old male with high-grade MDS bordering on acute myeloid leukemia on azacitidine and venetoclax presented with erythematous papulonodules of 3 weeks duration on his left thumb and right forearm unresponsive to 1 week of cephalexin (Fig 1, *A* and *B*). The patient denied fever, chills, and headache. A punch biopsy of the right forearm showed focal deep suppurative inflammation, including a mixed perivascular and interstitial inflammatory infiltrate with discrete foci of neutrophils, noted to be consistent with a skin or soft tissue infection, or alternatively a neutrophilic dermatosis (including Sweet's syndrome) if infection were ruled out. Grocott-Gomori methenamine silver (GMS), acid-fast bacilli (AFB), Fite, and Gram stains were negative for microorganisms. Concurrent punch biopsies for bacterial, fungal, and AFB cultures were also negative.

**Fig 2:**
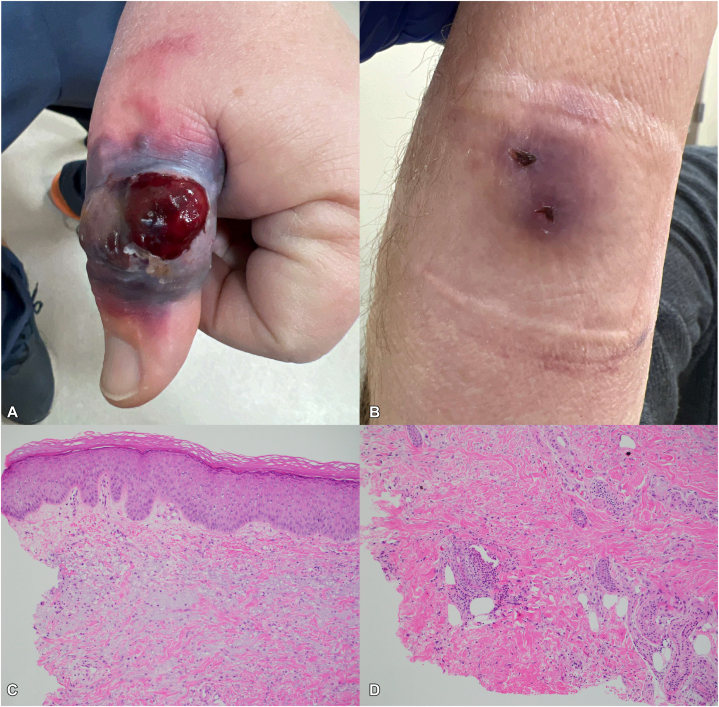
Progressive rash and resulting histology. Seven days after initial presentation, the physical exam was significant for **(A)** the left thumb with an edematous violaceous plaque and fungating hemorrhagic nodule and **(B)** the right forearm with a violaceous plaque and 2 hemorrhagic crusts from a previous biopsy. Histopathology of the second biopsy (from the left thumb) showed **(C)** mild perivascular and interstitial histiocytic infiltrate within the upper reticular dermis, along with **(D)** focal aggregates of prominent neutrophils in the deep dermis, consistent with Sweet's syndrome.

Seven days later, the left thumb nodule progressed to a large, painful, edematous, violaceous plaque with a central fungating, hemorrhagic nodule along the mediodorsal interphalangeal crease (Fig 2, *A*). A violaceous plaque with hemorrhagic crusting at the site of recent punch biopsy remained on the right forearm (Fig 2, *B*). An additional punch biopsy of the left thumb was performed for GMS, AFB, Fite, Gram, and hematoxylin and eosin tissue stains and bacterial, fungal, and AFB cultures. Given the rapid evolution of the thumb lesion in the setting of immunosuppression, the patient was admitted to the hospital for an infectious workup. The patient was febrile to 38.9 °C, erythrocyte sedimentation rate was 55 mm/hour (0-20 mm/hour), and C-reactive protein was 123 mg/L (0-8 mg/L). Blood cultures were negative. Empiric cefepime, vancomycin, metronidazole, and clindamycin were initiated given concern for infection. The second left thumb biopsy showed similar histopathology to the first. Tissue stains and cultures for microorganisms were again negative (Fig 2, *C* and *D*).

**Fig 3:**
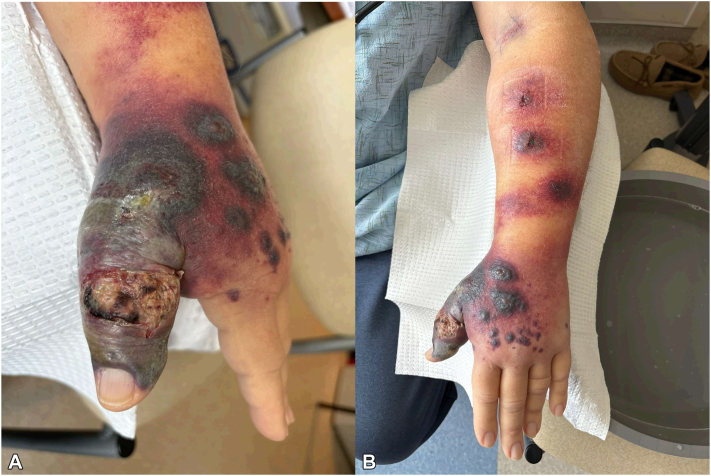
Sporotrichoid spread of nodules. The patient's **(A)** left hand with significant progression of lesion, now a violaceous plaque extending from the wrist down the thumb, with ulceration over the left thumb interphalangeal joint as well as multiple satellite, violaceous papules and nodules, some with overlying bullae, and **(B)** left forearm with erythematous, indurated nodules without ulceration following lymphatic drainage.

Three days after admission, the patient continued to experience intermittent fevers. The violaceous plaque now encompassed most of the left thumb, extending over the dorsal hand (Fig 3, *A*). Notably, there were new painful, erythematous, indurated nodules tracking up the left forearm in a sporotrichoid pattern (Fig 3, *B*), highly suspicious for a progressive deep fungal or mycobacterial infection, particularly in the context of immunosuppression. Given increasing concern for a yet unknown infectious etiology, biopsies of a new nodule on the left forearm were performed for tissue stains (hematoxylin and eosin, AFB, GMS, and Gram) as well as culture. Considering the rapidly progressive presentation, microorganisms would have likely been evident on tissue staining at this time if infectious. However, results were again negative for all tissue cultures and stains. Blood cultures remained negative.

**Fig 4:**
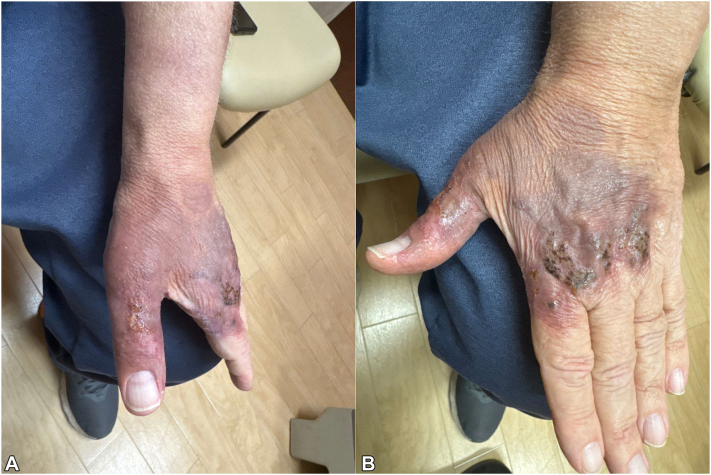
Resolution on steroids. Two and a half months after steroid initiation, the patient's **(A)** left dorsal hand showed markedly improved violaceous, thin plaques with minimal hemorrhagic crusting most prominent over the first 3 metacarpophalangeal joints and **(B)** the left thumb with confluent, violaceous thin plaque and a small focus of crusting over the proximal interphalangeal joint. No signs of necrosis were present.

The patient's cutaneous disease worsened, and fevers continued despite the use of broad antimicrobials. Since no infection had been elucidated from repeated blood cultures, tissue culture, or histology, and all 3 biopsies were consistent with Sweet's syndrome, IV methylprednisolone 1 mg/kg/day was initiated for treatment of suspected NDDH. The patient defervesced immediately with no fevers recorded after steroid initiation, pain and swelling improved, and no new nodules appeared. Antimicrobials were narrowed to vancomycin and cefepime without worsening of disease. After 4 days, IV methylprednisolone was tapered to 60 mg daily, and further tapered to oral prednisone 60 mg daily 3 days later. He was discharged on a 2-week course of doxycycline and ciprofloxacin with a prolonged prednisone taper. While he did experience a brief flare when the prednisone dose was initially decreased to 40 mg daily, the lesions have otherwise continued to improve without concomitant antibiotic therapy. He is currently at week 10 of his steroid course on prednisone 30 mg daily (Fig 4, *A* and *B*).

## Discussion

This case is consistent with the NDDH variant of Sweet's syndrome, meeting both major diagnostic criteria for Sweet's syndrome (abrupt onset of painful nodules or plaques and histology showing sterile, dense collection of neutrophils without evidence of vasculitis) and all 4 minor criteria (fever, a temporal relationship to malignancy, elevated inflammatory markers, and a positive response to corticosteroids).^2^^,^^6^ Despite meeting all diagnostic criteria, the presentation was highly suspicious for an infection associated with sporotrichoid spread, especially given his underlying malignancy and immunosuppression. However, infection was ruled out with repeated negative tissue cultures for bacteria, fungi, and AFB; numerous tissue stains without evidence of microorganisms; and failure to respond to broad-spectrum antibiotics. Unfortunately, the significant overlap in presentation between Sweet's syndrome and cutaneous infection can result in treatment delay while an infectious etiology is ruled out. The combination of clinical and histopathologic findings, rapid response to systemic steroids, and absence of infection confirms NDDH as the primary diagnosis in this case.

The presentation of NDDH in this patient was particularly unique due to the sporotrichoid spread of lesions, a distribution more commonly associated with infection. A literature search on PubMed, Embase, and Web of Science yielded only 1 documented case of Sweet's syndrome presenting with sporotrichoid spread. A 53-year-old woman with sore throat, fever, and an inflamed left wrist was treated with oral antibiotics for cellulitis, but remained febrile with worsening of the wrist lesion and new nodules spreading up the arm. Biopsy showed a neutrophilic dermatosis with negative bacterial, mycobacterial, and fungal cultures. Potassium iodide was initiated for Sweet's syndrome and within a few days the skin lesions and symptoms improved.^1^

Here, we present a rare case of NDDH with sporotrichoid spread in a 66-year-old male with MDS. This case emphasizes the diagnostic difficulty in distinguishing Sweet's syndrome from infection. Providers should not discount a diagnosis of Sweet's syndrome or NDDH in patients presenting with sporotrichoid lesions.

## Conflicts of interest

None disclosed.

## References

[1] Cohen P.R., Kurzrock R. (2000). Sweet's syndrome: a neutrophilic dermatosis classically associated with acute onset and fever. Clin Dermatol.

[2] Agrawal A., Arif S.H., Kumarasan K., Janjua D. (2022). Sweet's syndrome: an update. Curr Pediatr Rev.

[3] Paydas S. (2013). Sweet's syndrome: a revisit for hematologists and oncologists. Crit Rev Oncol Hematol.

[4] DiCaudo D.J., Connolly S.M. (2002). Neutrophilic dermatosis (pustular vasculitis) of the dorsal hands: a report of 7 cases and review of the literature. Arch Dermatol.

[5] Micallef D., Bonnici M., Pisani D., Boffa M.J. (2023). Neutrophilic dermatosis of the dorsal hands: a review of 123 cases. J Am Acad Dermatol.

[6] Orfaly V.E., Shakshouk H., Heath M., Hamilton A., Ortega-Loayza A.G. (2023). Sweet syndrome: a review of published cases. Dermatology.

